# The dual role of thrombospondin-1 in inflammatory regulation during acute respiratory distress syndrome: a mini-review

**DOI:** 10.3389/fimmu.2025.1699900

**Published:** 2025-12-19

**Authors:** YaWen Zheng, Cong Liu, YaJun Li, WenFei Wang, QingLi Dou

**Affiliations:** 1 Department of Emergency Medicine, The People’s Hospital of Baoan Shenzhen, Shenzhen, China; 2 Department of Emergency Intensive Care Unit, Shenzhen Baoan Air Sea Hospital, Shenzhen, China

**Keywords:** ARDS, COVID-19, cytokines, infection, inflammatory, Thbs1/TSP1

## Abstract

Inflammation serves as a fundamental defense against tissue injury and infection, yet dysregulation can lead to pathological outcomes. Thrombospondin-1 (Thbs1/TSP1), a multifunctional glycoprotein significantly upregulated during inflammation, exemplifies a dualistic regulator with context-dependent roles. Through modulation of cytokine networks and inflammatory cell activity (notably macrophages), Thbs1 critically governs inflammatory responses. Acute respiratory distress syndrome (ARDS), a life-threatening condition fueled by systemic inflammation secondary to infection or trauma, presents complex pathophysiology requiring elucidation. COVID-19 research highlights elevated Thbs1 expression in severe patients, where it demonstrates protective effects against pulmonary damage primarily via extracellular matrix protection, inhibition of neutrophil serine proteases, and TGF-β-dependent repair pathways. However, paradoxical evidence indicates that dysregulated Thbs1 can also contribute to ARDS pathogenesis, potentially by amplifying inflammation, promoting thromboinflammation, or driving fibrosis. Mechanistic insights reveal Thbs1’s influence on ARDS progression through ECM remodeling, serine protease inhibition, and TGF-β activation. While significant progress has been made in understanding Thbs1 signaling, the precise mechanisms dictating its context-dependent switch between protective and pathogenic functions in inflammatory pathways remain a critical area for future investigation.

## 1 Introduction

Thrombospondin-1 (Thbs1/TSP1), a trimeric glycoprotein, is a multifunctional regulator initially identified as an angiogenesis inhibitor. Its modular domains (N-terminal, TSR1–3 repeats, C-terminal) enable interactions with heparin, integrins, CD36, CD47, and extracellular matrix components, influencing angiogenesis, inflammation, tumor biology, and immune regulation (1–7). Primarily released from platelet α-granules upon activation, Thbs1 is also secreted by macrophages, endothelial cells, and epithelial cells (8, 9). In inflammatory diseases, Thbs1 exhibits context-dependent roles: it promotes neutrophil recruitment, macrophage phagocytosis, and cytokine production (TNF-α, IL-6) via CD36/TLR4/NF-κB pathways, yet it also resolves inflammation by inducing IL-10 production and apoptotic cell clearance (10–13). Critically, Thbs1 is upregulated in acute respiratory distress syndrome (ARDS) and coronavirus disease 2019 (COVID-19), where it influences extracellular matrix integrity, neutrophil serine protease activity, TGF-β signaling, and tissue repair (14–18). Despite its protective roles against pathogen-induced lung injury, dysregulated Thbs1 contributes to immune hyperactivation and tissue damage in ARDS pathogenesis (14, 19–27).

## 2 Methods

This mini-review synthesizes preclinical and clinical evidence on Thbs1’s roles in inflammation and ARDS from January 2020 to June 2025. We searched PubMed utilizing the following search terms: ARDS AND Thbs1/TSP1, with the most recent search conducted until June 2025.

### 2.1 Inclusion and exclusion criteria

Studies were included if they reported specifically on adult inflammatory disorders (atherosclerosis, colitis, ARDS), infectious complications (*Pseudomonas*, *Klebsiella* infections), COVID-19 pathogenesis, and Thbs1’s signaling pathways. References from studies that had their full text reviewed but did not meet the inclusion criteria were searched to identify any missed literature, with resultant abstracts reviewed utilizing the same inclusion criteria above. Studies were excluded if they did not report on the inflammatory roles of Thbs1.

## 3 The dual faces of Thbs1 in inflammation and immunity

Thbs1 exemplifies a matricellular protein with context-dependent functions in inflammation (28, 29). Its ability to either promote or resolve inflammatory processes hinges on the cellular milieu, receptor engagement, and disease stage (30–32).

### 3.1 Pro-inflammatory actions

Thbs1 can exacerbate inflammation by enhancing leukocyte recruitment and pro-inflammatory cytokine production. It interacts with CD36 on macrophages to potentiate TLR4/NF-κB signaling, increasing TNF-α and IL-6 expression (11, 33–35). In obesity models, elevated Thbs1 contributes to macrophage activation and metabolic dysfunction (35). Thbs1 also promotes the adhesion and migration of monocytes, although its role in atherosclerosis remains complex and contested (36–38). In severe infections or tissue damage, dysregulated Thbs1 can contribute to neutrophil hyperactivation and tissue injury (19–21, 39–41).

### 3.2 Anti-inflammatory and resolution actions

Conversely, Thbs1 is crucial for resolving inflammation. It facilitates the clearance of apoptotic cells via CD36, triggering macrophage production of the anti-inflammatory cytokine IL-10 (10, 42, 43). Thbs1 deficiency impairs this process, leading to defective IL-10 production and exacerbated lung injury (10). In models of bacterial infection (e.g., *Klebsiella pneumoniae*), Thbs1 restrains neutrophil serine protease activity (NE, CG), preventing excessive tissue damage and promoting host survival (14, 19, 44–46). Furthermore, Thbs1 can activate latent TGF-β, a cytokine involved in immunosuppression and tissue repair (47, 48).

## 4 Thbs1 in ARDS and COVID-19: a prototypical dual role

ARDS, characterized by diffuse alveolar damage and uncontrolled inflammation, represents a clinical scenario where Thbs1’s dual role is critically relevant (49, 50). The COVID-19 pandemic provided further insights, revealing significant Thbs1 upregulation in severe patients (16–18, 51–53).

### 4.1 Protective mechanisms

Thbs1 protects against lung injury primarily by 1) extracellular matrix (ECM) protection: Thbs1 inhibits bacterial metalloproteinases (*Pseudomonas aeruginosa* LasB), safeguarding ECM proteins from degradation and reducing lung permeability (6, 14, 54, 55). 2) Neutrophil serine protease regulation: It acts as a competitive inhibitor of neutrophil-derived elastase (NE) and cathepsin G (CG), curtailing their destructive potential when released extracellularly (15, 56, 57). Thbs1 deficiency leads to unchecked protease activity, worsened lung damage, and increased mortality in some infection models (58, 59). 3) Repair and resolution: Thbs1 secreted from endothelial cells or platelets can promote the differentiation of bronchoalveolar stem cells into alveolar type II cells, aiding repair after injury (22, 31, 60). Its role in activating TGF-β and facilitating apoptotic cell clearance further supports inflammation resolution (10, 47).

### 4.2 Pathogenic potential

In murine models of ARDS, TSP1 contributes to thromboinflammation but exhibits protective effects in repair pathways (34). Persistent Thbs1-mediated TGF-β activation might also drive fibrotic progression in late-stage ARDS (61).

Crucially, studies in COVID-19 patients reveal TSP1-specific pathophysiological alterations distinct from ARDS. Elevated TSP1 levels in severe cases significantly correlate with increased mortality risk, while its direct interaction with viral structural proteins exacerbates thromboinflammation (62, 63). Spatial histopathological analyses further demonstrate concentrated TSP1 overexpression within fibrotic foci of post-COVID lungs (64). These findings collectively underscore TSP1’s unique role in COVID-19 pathogenesis through 1) prognostic biomarker utility, 2) virus–protein interaction-driven thrombosis, and 3) localized fibrotic remodeling. This disease-specific pathophysiology—summarized in **Table 1**—establishes TSP1 as a critical mediator beyond generalized ARDS mechanisms.

**Table 1:** Context-dependent roles of Thbs1 in inflammation and ARDS.

Context/condition	Pro-inflammatory/detrimental effects	Anti-inflammatory/protective effects	References
Immune cell activation signaling	Promotes neutrophil recruitment, macrophage phagocytosis, and pro-inflammatory cytokine (TNF-α, IL-6) production via CD36/TLR4/NF-κB pathways.	Facilitates apoptotic cell clearance via CD36, triggering macrophage IL-10 production to resolve inflammation. Restrains neutrophil serine protease (NE, CG) activity.	(11, 34)
Infectious models (Thbs1^+^/^−^ mice)	Contributes to hyperinflammation and tissue damage (e.g., *Pseudomonas* infection model).	Protects against ECM degradation; restrains neutrophil serine protease activity; improves bacterial clearance.	(20, 21)
Sterile injury	Dysregulated expression may contribute to chronic inflammation and fibrosis.	Drives stem cell differentiation for repair; deficiency leads to worse lung injury.	(29, 64)
Metabolic inflammation	Elevated expression contributes to macrophage activation, adipose inflammation, and metabolic dysfunction in obesity.	Deficiency reduces obesity-associated inflammation and improves insulin sensitivity.	(35)
Vascular biology/atherosclerosis	Promotes monocyte adhesion and migration. Enhances macrophage infiltration *in vitro*. Expression is elevated in aneurysm tissue.	Deficiency in ApoE^+^/^−^ mice accelerates atherosclerotic plaque maturation (indicating a protective role under wild-type conditions).	(36, 37)
ARDS (patient data and models)	High levels correlate with injury severity scores; may contribute to thromboinflammation and cytokine amplification and might drive fibrotic progression in late-stage ARDS via persistent Thbs1-mediated TGF-β activation (suggestive, potential role).	Elevated thrombospondin-1 levels are associated with protective effects in patient studies/experimental models; mediates ECM protection, limits proteolysis, and promotes repair.	(34, 61)
COVID-19 (severe patients)	Identified alterations: upregulated expression correlates with severity. Contributes to persistent TGF-β signaling driving fibrosis; strong association with thromboinflammation and hypercytokinemia.	Elevated thrombospondin-1 levels are associated with protective effects in subsets; mediates ECM protection and limits proteolysis (note: protective effects may occur concurrently or in different contexts/pathways).	(62–64)

## 5 Therapeutic implications and future directions

The duality of Thbs1 makes it a challenging yet intriguing therapeutic target. Strategies could aim to inhibit its pro-inflammatory interactions or augment its protective functions in a context-specific and temporal manner. The key questions for future research may be the specific cues to determine whether Thbs1 acts as a pro-inflammatory or anti-resolution signal in the human lung.

## 6 Limitations

This review has limitations. Firstly, the literature search was restricted to PubMed and the English language, potentially omitting relevant studies. Secondly, the mechanistic evidence is largely derived from preclinical models; direct validation in human ARDS pathophysiology is needed.

## 7 Conclusion

**Figure 1:**
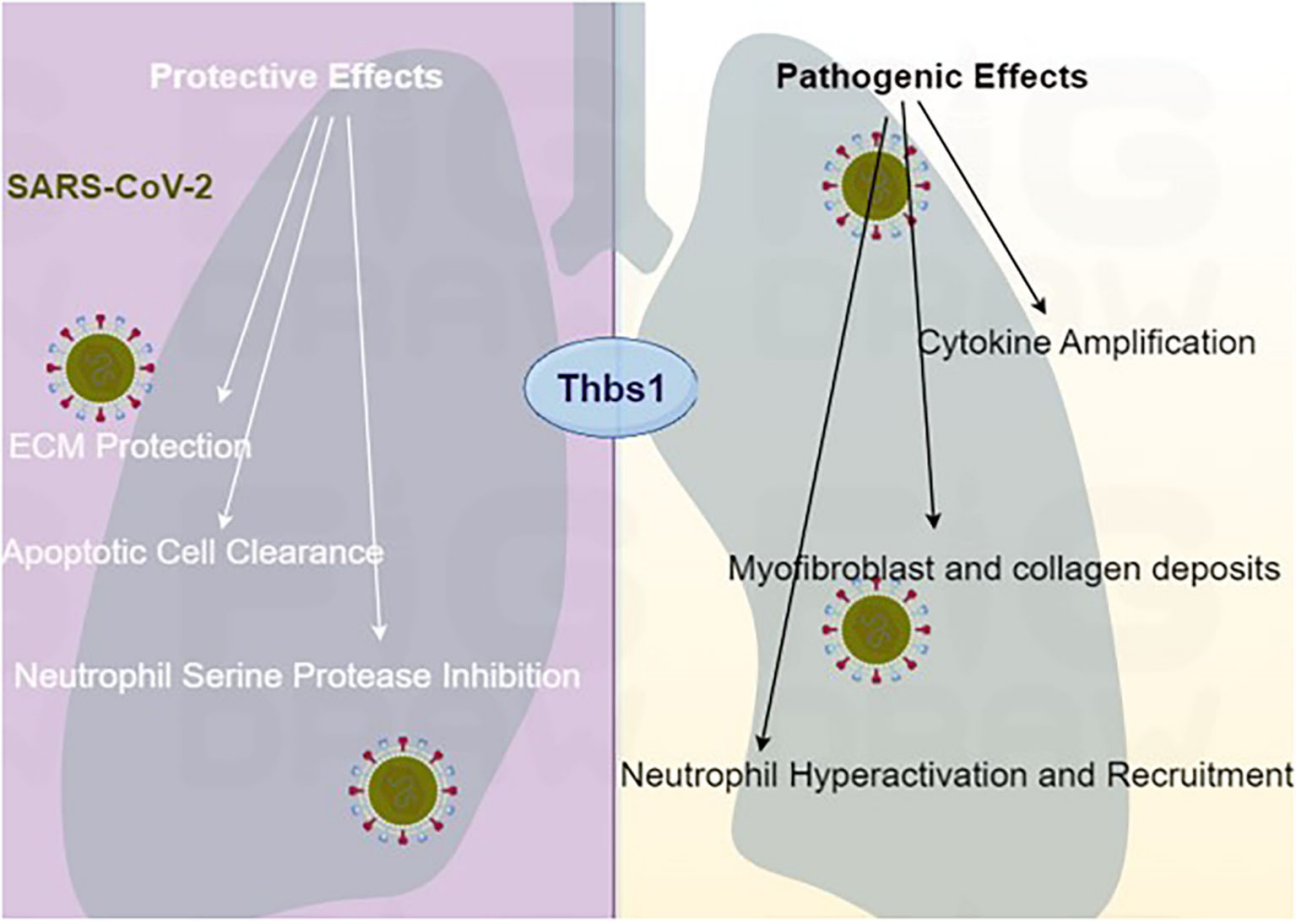
Proposed dual roles of Thbs1 in ARDS pathogenesis.

Thbs1 is a quintessential example of a matricellular protein with opposing functions in inflammation and ARDS. In COVID-19-associated ARDS, it largely exerts protective effects against lung damage, but its dysregulation can contribute to pathology (**Figure 1**). Understanding the mechanisms that dictate this balance, such as specific receptor usage, proteolytic processing, and temporal expression, is paramount. Future research must move beyond descriptive studies toward mechanistic dissection and therapeutic modulation of specific Thbs1 pathways, offering hope for novel treatments in ARDS and other inflammatory conditions.
